# Treatment of Primary Pulmonary Synovial Sarcoma in a Low-Resource Country: A Case Report

**DOI:** 10.7759/cureus.79463

**Published:** 2025-02-22

**Authors:** Araksi Gasparyan, Tatul Saghatelyan, Sergey Abrahamyan, Narek Shaverdian, Sergo Mkhitaryan

**Affiliations:** 1 Department of Oncology, Yerevan State Medical University, Yerevan, ARM; 2 Department of Radiation Oncology, National Center of Oncology, Yerevan, ARM; 3 Department of Thoracic Surgery, National Center of Oncology, Yerevan, ARM; 4 Department of Radiation Oncology, Memorial Sloan Kettering Cancer Center, New York City, USA

**Keywords:** adjuvant radiotherapy, case report, lung cancer, synovial sarcoma, thoracic surgery

## Abstract

Primary synovial sarcoma of the lung is a rare malignant cancer. The symptoms are nonspecific; they may not appear at all, so it is often diagnosed in the late stages. Moreover, accurate diagnosis requires a combination of the results of clinical evaluation, imaging, and histological examinations, since the results of these methods separately are not very specific and may lead to misdiagnosis. Given the rarity of this diagnosis, the therapy is not standardized. The preferred treatment regimen involves surgery combined with neoadjuvant or adjuvant radio- and chemotherapy. This case report describes a 67-year-old male patient who was diagnosed with pulmonary synovial sarcoma. Diagnosis was confirmed via computed tomography (CT) imaging and immunohistochemical (IHC) examination. After surgical removal, the patient underwent adjuvant radiotherapy.

## Introduction

Primary pulmonary synovial sarcoma (PPSS) is a rare malignant tumor accounting for less than 0.5% of all lung tumors [1]. It mostly originates in the parenchyma of the lung; however, it can arise in the pleura and mediastinum as well [1,2]. PPSS is very aggressive, and clinical outcomes have remained poor [1].

The specific cell of origin for PPSS is yet to be discovered. Common histological subtypes of synovial sarcomas are the biphasic histological subtype, consisting of epithelial and spindle cells, and the monophasic histological subtype, composed of spindle cells [3]. Previously reported cases of PPSS have mainly described monophasic tumors [4].

Common symptoms include chest pain, cough, and hemoptysis, although published series suggest a substantial portion of patients can be asymptomatic [4]. The diagnosis can be established through a combination of clinical evaluation, imaging, histological features, and immunohistochemistry findings to differentiate it from other primary lung tumors and metastatic sarcomas [5]. The lack of literature and protocols regarding this diagnosis results in the lack of a defined standardized treatment plan. As a preferred treatment plan, patients undergo surgery, which is frequently accompanied by neoadjuvant or adjuvant radiation therapy and chemotherapy [4]. In this report, we present a case of synovial sarcoma of the lung in a 67-year-old male patient who underwent radiotherapy after surgical resection of the tumor.

## Case presentation

Main complaints

A 67-year-old male patient was referred to the radiotherapy department for further discussion on adjuvant therapies. During the consultation, the patient did not report any focal complaints. The only complaint that was mentioned was modest pain in the right chest wall connected with surgery. The patient is a non-smoker, the Eastern Cooperative Oncology Group (ECOG) status was evaluated and was estimated as grade 1.

The clinical course of diagnosis

**Figure 1:**
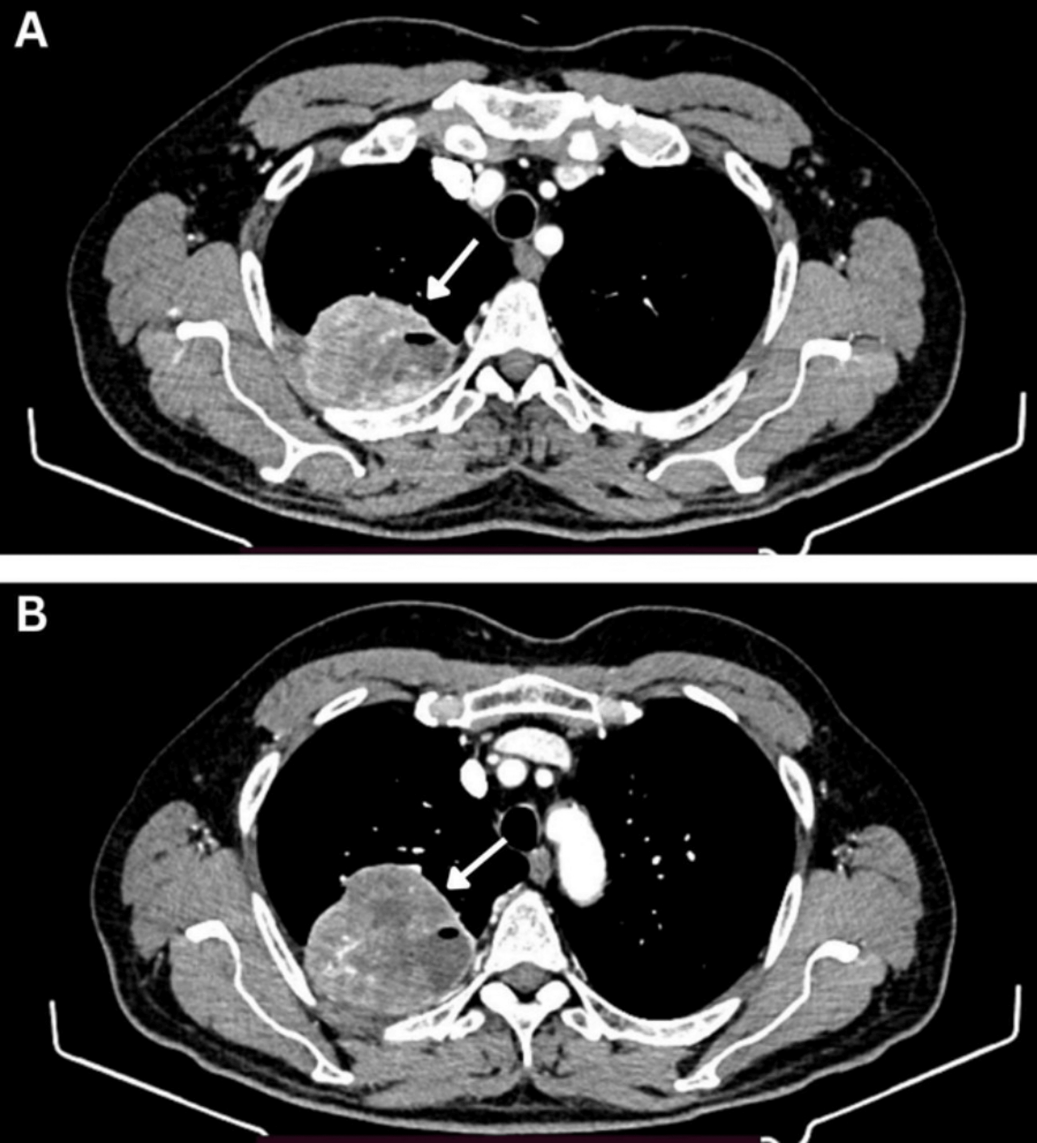
A and B: the solid-cystic formation with clear edges measuring 7.0x7.6x6.9 cm (arrow) was found in the upper lobe of the right lung on May 18, 2022.

The main presenting symptoms started two years prior when the patient experienced chest pain, cough, wheezing, and shortness of breath. On May 18, 2022, a computed tomography (CT) was performed. CT imaging revealed a solid-cystic formation with well-defined edges measuring 7.0x7.6x6.9 cm located peripherally in the S1 segment of the upper lobe of the right lung (Figures 1A, 1B). No visible ingrowth into the posterior chest wall or paracostal pleura was found. Secondary pathological changes in other organs were not described.

The patient applied to the Department of Thoracic Surgery and underwent surgery on May 26, 2022. Right thoracotomy, upper lobectomy combined with dissection of the chest wall (II-III ribs), and mediastinal lymph node dissection were performed. Pathohistological and immunohistochemical (IHC) examinations revealed the diagnosis: primary synovial sarcoma of the upper lobe of the right lung, pT4 (multifocal involvement), pN0 (0/35), cM0, L0, V0, pn0, R0. The results of the identification of IHC markers are shown below (Table 1).

**Table 1:** The results of detection of immunohistochemical markers. PanCK: Pan cytokeratin, CD56: Neural cell adhesion molecule, CD45: Lymphocyte common antigen, SMA: Spinal muscular atrophy, TLE1: Transducin-like enhancer of split 1, CD99: Single-chain type-1 glycoprotein, BCL2: B-cell leukemia/lymphoma 2 protein, CD15: Carbohydrate-based molecule, CD30: Activation antigen, S100: S100 protein, Ki67: Ki67 protein, CD34: transmembrane phosphoglycoprotein protein, CD20: CD20 protein

Marker	Presence in tumor tissue
PanCK	Negative
Vimentin	Positive
S100	Negative
CD45	Negative
Ki67	80-85%
CD34	Negative
CD56	Positive
SMA	Weakly positive
Desmin	Negative
TLE1	Weakly positive
CD99	Negative
BCL2	Negative
CD20	Negative
CD15	Negative
CD30	Negative

The upper lobe of the right lung with resection of the chest wall and II-III ribs was presented for examination. The lung tissue was 16.0x12.0x6.0 cm in volume, in which a mass with cystic degeneration with the size of 8.0x8.5x8.0 cm was found, with spots of necrosis and hemorrhage in the lobes. The mass was characterized by endo- and peribronchial growth, located at a distance of 4.0 cm from the hilum. There is a macroscopically visible ingrowth in pleural sheets, soft tissues of the chest wall, and ribs. The tumor grade is G3, and there was no lymphovascular invasion (L0, V0). However, there was no information on whether there was a close margin while resecting the tumor.

Family history

No significant findings were obtained.

Laboratory tests

The results of laboratory tests are shown in Table 2.

**Table 2:** Laboratory test results. WBC: White blood cells, RBC: Red blood cells, HGB: Hemoglobin, PLT: Platelet count, AST: Aspartate transaminase, ALT: Alanine transaminase, Na+: Sodium, K+: Potassium, Ca2+: Calcium ions, INR: International normalized ratio

Lab test	Result	Normal range
WBC	4.05 x 10^3^/μl	4.5-11.0 x 10^3^/μl
RBC	5.41 x 10^6^/μl	4.7-6.1 x 10^6^/μl
HGB	155 g/L	138-172 g/L
PLT	184 x 10^3^/μl	150-400 x 10^3^/μl
AST	25.2 U/L	8-33 U/L
ALT	24.4 U/L	4-36 U/L
Creatinine	71.1 mmol/L	65.4-119.3 mmol/L
Bilirubin	15.3 mmol/L	3-17 mmol/L
Na^+^	143 mmol/L	136-145 mmol/L
K^+^	4.6 mmol/L	3.6-5.2 mmol/L
Ca^2^^+^	1.12 mmol/L	1.05-1.13 mmol/L
Prothrombin time	13.6 seconds	11-13.5 seconds
Prothrombin index	86.0%	> 70%
INR	1.2	< 1.1
Fibrinogen	355 mg/dL	200-400 mg/dL

Imaging results

**Figure 2:**
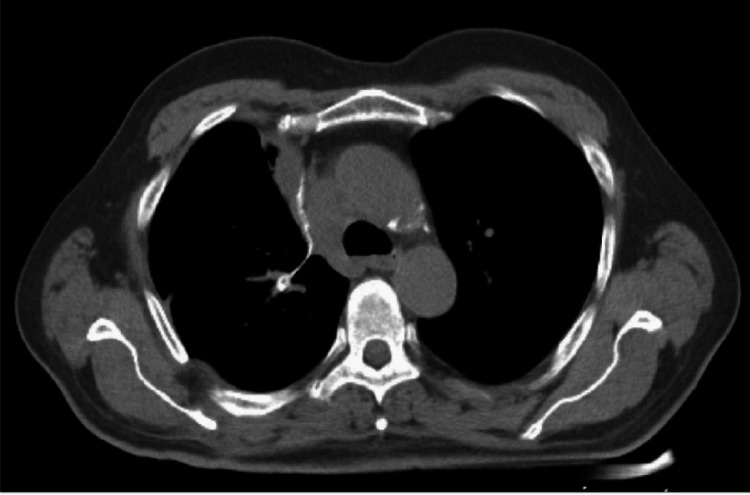
Postoperative CT image: no gross residual tumor was identified in the lung.

CT simulation of the chest performed on August 8, 2022, showed no gross residual tumor tissue (Figure 2). An ultrasound of the abdomen and pelvis performed on August 9, 2022, revealed no secondary pathological changes.

Radiation treatment

**Figure 3:**
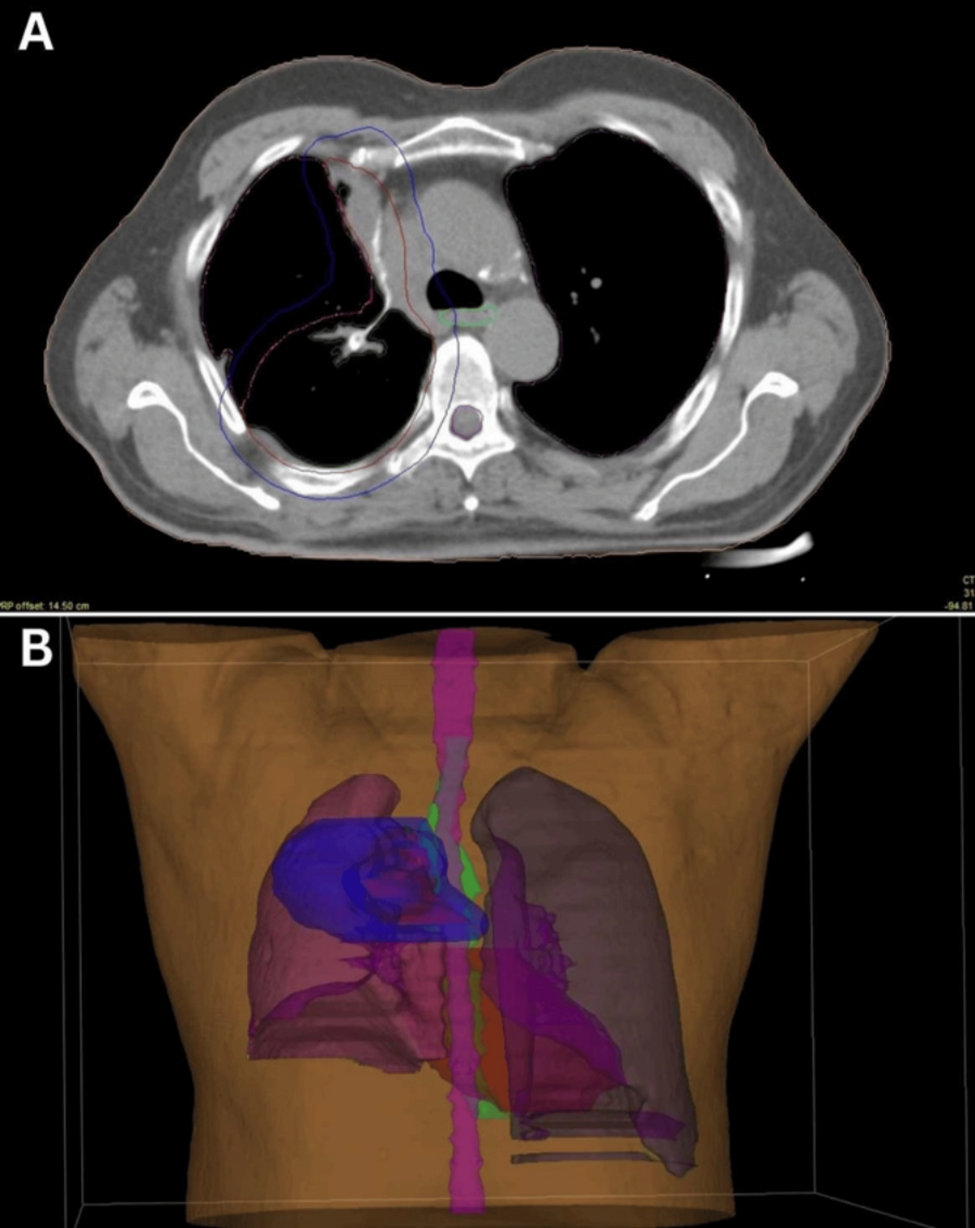
A: CT simulation image of the chest with radiation volumes. B: 3D reconstruction of the irradiation field mentioned in Figure 3A. A: Organs at risk included the esophagus (light green curve), spinal cord (purple curve), heart, and both lungs (green curves). The clinical target volume (CTV, red curve) was derived based on pre-operative imaging and surgical pathology and included at-risk suture and adjacent lung parenchyma. Expansion from CTV to planning target volume (PTV, blue curve) was 1 cm.

Given the rarity of the diagnosis, there are currently no specific guidelines based on level-one evidence. Treatment was based on published research findings and individual case reports [1,3,6]. The patient was prescribed 30 fractions of adjuvant three-dimensional conformal radiation therapy (3D-CRT) to a total dose of 60 Gy in 2 Gy fractions. The irradiation plan is shown in Figure 3. All these curves were contoured according to Radiation Therapy Oncology Group (RTOG) atlases and guidelines [7]. 3D conformal radiotherapy was delivered using a 6 MEV ELEKTA linear accelerator irradiating the area of the at-risk post-surgical volume. The treatment started on August 23, 2022, and proceeded smoothly; no complications were observed. On October 7, 2022, the patient's treatment was completed, and he was discharged in satisfactory condition.

Follow-up

After the end of adjuvant radiotherapy, whole-body CT imaging was performed every three months. The patient presented mild dysphagia, which, however, did not require any treatment. He underwent routine laboratory tests and CTs once every three months. With a follow-up of two years, there is no evidence of disease recurrence.

## Discussion

Out of all lung tumors, primary pulmonary synovial sarcoma accounts for less than 0.5% [1]. The tumor usually occurs in older patients, regardless of gender [4]. The main symptoms include dyspnea, chest pain, hemoptysis, and cough; however, on some occasions, it can be found incidentally [4]. In our case, the patient is a 67-year-old male who reported chest pain, wheezing, shortness of breath, and cough.

To establish a proper diagnosis, the tumor is primarily identified using either CT or magnetic resonance imaging (MRI) [8]. Primary synovial sarcomas are described as well-circumscribed and heterogeneous, being either solid or cystic-solid masses when imaging [8]. Additional features may include perilesional edemas, extension to the neighboring tissues, intratumoral hemorrhage, and calcification [8]. In our case, the tumor was located peripherally, had well-circumscribed borders, and was of a cystic-solid formation. No secondary and peritumoral changes were seen.

After identification, the exact histological type of the tumor must be confirmed. The origin of the cells forming PPSS remains unclear. Morphologically, it can be of four categories, from which the two major types include the monophasic fibrous type, consisting of fibroblast-like spindle cells, and the biphasic type, consisting of both spindle and epithelial cells [2,9]. The rare types of sarcomas include the monophasic epithelial type and the poorly differentiated type [9]. Histological and immunohistochemical examinations are used to identify the type of synovial sarcoma. Markers that are commonly expressed on synovial sarcoma cells include epithelial membrane antigen (EMA) [10], BCL-2 [11], and vimentin [12], while markers such as protein S-100 [12], CD99 [6], CD34 [6], actin [12], and desmin [12] are mostly negative. The results in our case correspond to this, although BCL-2 was found negative, and EMA was not identified. However, there is not a single marker that will be specific for PPSS, and even in combination, IHC markers may not definitively confirm or exclude the presence of the tumor [13]. In such cases, the IHC should be reviewed and interpreted, taking into account the clinical presentation and the imaging results. Synovial sarcomas present a highly specific translocation t(X;18)(p11.2;q11.2) [14]. The SS18 gene fuses with SSX genes, resulting in the generation of fusion oncogenes, which are the main drivers of the synovial sarcoma's pathophysiology [14]. Hence, examinations like fluorescence in situ hybridization (FISH) and reverse transcription polymerase chain reaction (RT-PCR) are more specific and considered one of the "gold standards" in diagnosis [15]. However, due to the high cost and time requirements, combined examinations with imaging and histological and IHC tests are commonly preferred. Considering the fact this genetic test was not available in Armenia, the diagnosis was made by combining the information from imaging, histological, and IHC tests.

Given the rarity of the diagnosis, there is no standardized therapy. Whenever possible, surgery is recommended together with adjuvant and/or neoadjuvant chemotherapy and/or radiotherapy [4,6]. Lobectomy and pneumonectomy are suggested surgeries combined with the removal of enlarged mediastinal nodes; in the case of a small lesion tumorectomy, it might be considered [2]. Lobectomy followed by irradiation is especially recommended for local, high-grade (G2-G3), extensive lesions bigger than 5 cm [16]. Commonly, the general dose range is 54-60 Gy, fractionated into 1.8-2 Gy daily [16]. The dose can be increased up to 70 Gy if there is a concern for non-R0 margins. The dosage was chosen based on the available case reports and lung cancer radiotherapy guidelines [7,17,18]. Since the patient had a large lesion and there was no visible ingrowth into neighboring tissues on the CT scan image, lobectomy with the removal of mediastinal lymph nodes was performed, followed by adjuvant radiotherapy.

Common toxicities of lung radiotherapy include radiation pneumonitis with further lung fibrosis and respiratory insufficiency, dysphagia, and odynophagia with obstruction of the esophagus, in severe cases with further ulceration and perforation [19]. In our case, mild dysphagia was clinically observed, which did not require special treatment. The patient underwent routine laboratory tests and CTs, and post-operative and post-radiation changes in the right lung were described during subsequent CTs. As of now, no other toxicities have been observed.

PPSS is an unpredictable tumor, reaching up to 50% five-year overall survival [20]. Therefore, follow-up after the treatment and regular check-ups are crucial. As for our patient, no relapse occurred over two years.

The main limitations over the course of treatment were the lack of an initial biopsy and the absence of an SS18:SSX gene fusion testing opportunity in Armenia. The surgery was done considering the results of the CT scan. The final diagnosis was made based on collective information derived from a CT scan, histological, and IHC findings.

## Conclusions

The patient presented with non-specific symptoms implying lung disease. Imaging results confirmed the presence of solid formation in the lung, which was further histologically confirmed to be pleuropulmonary synovial sarcoma. Since there was not any visible ingrowth into neighboring tissues on CT scan images, lobectomy with removal of lymph nodes was performed. Considering that the tumor is unpredictable and aggressive, adjuvant radiotherapy was performed. After a two-year follow-up, disease recurrence was not observed.

This article presents a case of rarely diagnosed cancer to help in collecting information on available diagnostic methods and treatments, as the literature on this topic is scarce. Moreover, it highlights the main challenges the low-resource countries meet while organizing diagnostic and treatment plans, for instance, the lack of an initial biopsy or the absence of genetic testing to find the gene transfusion specific to the disease.

## References

[1] Etienne-Mastroianni B, Falchero L, Chalabreysse L (2002). Primary sarcomas of the lung: a clinicopathologic study of 12 cases. Lun Can.

[2] Panigrahi MK, Pradhan G, Sahoo N (2018). Primary pulmonary synovial sarcoma: a reappraisal. J Cancer Res Ther.

[3] Cummings NM, Desai S, Thway K (2010). Cystic primary pulmonary synovial sarcoma presenting as recurrent pneumothorax: report of 4 cases. Am J Surg Pathol.

[4] Zeren H, Moran CA, Suster S (1995). Primary pulmonary sarcomas with features of monophasic synovial sarcoma: a clinicopathological, immunohistochemical, and ultrastructural study of 25 cases. Hum Pathol.

[5] Kumar R, Menon S, Desai SB (2009). Primary endobronchial synovial sarcoma confirmed by SYT-SSX1 fusion gene transcript by reverse transcriptase polymerase chain reaction. Indian J Pathol Microbiol.

[6] Lan T, Chen H, Xiong B (2016). Primary pleuropulmonary and mediastinal synovial sarcoma: a clinicopathologic and molecular study of 26 genetically confirmed cases in the largest institution of southwest China. Diagn Pathol.

[7] Kong FM, Ritter T, Quint DJ (2011). Consideration of dose limits for organs at risk of thoracic radiotherapy: atlas for lung, proximal bronchial tree, esophagus, spinal cord, ribs, and brachial plexus. Int J Radiat Oncol Biol Phys.

[8] Baheti AD, Tirumani SH, Sewatkar R (2015). Imaging features of primary and metastatic extremity synovial sarcoma: a single institute experience of 78 patients. Br J Radiol.

[9] Kottu R, Prayaga AK (2010). Synovial sarcoma with relevant immunocytochemistry and special emphasis on the monophasic fibrous variant. J Cytol.

[10] Fisher C (1986). Synovial sarcoma: ultrastructural and immunohistochemical features of epithelial differentiation in monophasic and biphasic tumors. Hum Pathol.

[11] Machen SK, Fisher C, Gautam RS (1998). Utility of cytokeratin subsets for distinguishing poorly differentiated synovial sarcoma from peripheral primitive neuroectodermal tumour. Histopathology.

[12] Ordóñez NG, Mahfouz SM, Mackay B (1990). Synovial sarcoma: an immunohistochemical and ultrastructural study. Hum Pathol.

[13] Zaborowski M, Vargas AC, Pulvers J (2020). When used together SS18-SSX fusion-specific and SSX C-terminus immunohistochemistry are highly specific and sensitive for the diagnosis of synovial sarcoma and can replace FISH or molecular testing in most cases. Histopathology.

[14] Crew AJ, Clark J, Fisher C (1995). Fusion of SYT to two genes, SSX1 and SSX2, encoding proteins with homology to the Kruppel-associated box in human synovial sarcoma. EMBO J.

[15] Fiore M, Sambri A, Spinnato P (2021). The biology of synovial sarcoma: state-of-the-art and future perspectives. Curr Treat Options Oncol.

[16] Kalpathi K, Linga VG, Digumarti R (2013). Primary pleuropulmonary synovial sarcoma: A report of two cases and review of literature. South Asian J Cancer.

[17] Rodrigues G, Choy H, Bradley J (2015). Definitive radiation therapy in locally advanced non-small cell lung cancer: Executive summary of an American Society for Radiation Oncology (ASTRO) evidence-based clinical practice guideline. Pract Radiat Oncol.

[18] Nestle U, De Ruysscher D, Ricardi U (2018). ESTRO ACROP guidelines for target volume definition in the treatment of locally advanced non-small cell lung cancer. Radiother Oncol.

[19] Bradley J, Graham MV, Winter K (2005). Toxicity and outcome results of RTOG 9311: a phase I-II dose-escalation study using three-dimensional conformal radiotherapy in patients with inoperable non-small-cell lung carcinoma. Int J Radiat Oncol Biol Phys.

[20] Frazier AA, Franks TJ, Pugatch RD, Galvin JR (2006). From the archives of the AFIP: Pleuropulmonary synovial sarcoma. Radiographics.

